# Neuroprotective potentials of selected natural edible oils using enzyme inhibitory, kinetic and simulation approaches

**DOI:** 10.1186/s12906-021-03420-0

**Published:** 2021-10-02

**Authors:** Mater H. Mahnashi, Bandar A. Alyami, Yahya S. Alqahtani, Ali O. Alqarni, Muhammad Saeed Jan, Muhammad Ayaz, Farhat Ullah, Muhammad Shahid, Umer Rashid, Abdul Sadiq

**Affiliations:** 1 Department of Pharmaceutical Chemistry, College of Pharmacy, Najran University, Najran, Saudi Arabia; 2 Department of Pharmacy, University of Swabi, Swabi, KP Pakistan; 3 Department of Pharmacy, Faculty of Biological Sciences, University of Malakand, Chakdara, KP 18000 Dir (L) Pakistan; 4 Department of Pharmacy, Sarhad University of Science and Information Technology, Peshawar, Khyber Pakhtunkhwa 25000 Pakistan; 5 Department of Chemistry, COMSATS University Islamabad, Abbottabad Campus, Abbottabad, 22060 Pakistan

**Keywords:** Functional foods, Free radicals, Cholinesterase’s, Alzheimer’s disease, Ginger, Cumin, Cinnamon and GC-MS

## Abstract

**Background:**

Edible oils have proven health benefits in the prevention and treatment of various disorders since the establishment of human era. This study was aimed to appraise neuropharmacological studies on the commonly used edible oils including *Cinnamomum verum* (CV)*, Zingiber officinale* (ZO) and *Cuminum cyminum* (CC).

**Methods:**

The oils were analyzed via GC-MS for identifications of bioactive compounds. Anti-radicals capacity of the oils were evaluated via 2,2-diphenyl-1-picryl-hydrazyl-hydrate (DPPH) and 2,2'-azino-bis(3-ethylbenzothiazoline-6-sulfonic acid (ABTS) radicals scavenging assays. The samples were also tested against two important acetylcholinesterase (AChE) and butyrylcholinesterase (BChE) which are among the important drug targets in Alzheimer’s disease. Lineweaver-Burk plots were constructed for enzyme inhibition studies which correspond to velocity of enzymes (V_max_) against the reciprocal of substrate concentration (*K*_*m*_) in the presence of test samples and control drugs following Michaelis-Menten kinetics. Docking studies on AChE target were also carried out using Molecular Operating Environment (MOE 2016.0802) software.

**Results:**

(Gas chromatography-mass spectrometry GC-MS) analysis revealed the presence of thirty-four compounds in Cinnamon oil (Cv.Eo), fourteen in ginger oil (Zo.Eo) and fifty-six in cumin oil (Cc.Eo). In the antioxidant assays, Cv.Eo*,* Zo.Eo and Cc.Eo exhibited IC_50_ values of 85, 121, 280 μg/ml sequentially against DPPH radicals. Whereas, in ABTS assay, Cv.Eo*,* Zo.Eo and Cc.Eo showed considerable anti-radicals potentials with IC_50_ values of 93, 77 and 271 μg/ml respectively. Furthermore, Cv.Eo was highly active against AChE enzyme with IC_50_ of 21 μg/ml. Zo.Eo and Cc.Eo exhibited considerable inhibitory activities against AChE with IC_50_ values of 88 and 198 μg/ml respectively. In BChE assay, Cv.Eo*,* Zo.Eo and Cc.Eo exhibited IC_50_ values of 106, 101 and 37 μg/ml respectively. Our results revealed that these oils possess considerable antioxidant and cholinesterase inhibitory potentials. As functional foods these oils can be effective remedy for the prevention and management of neurological disorders including AD. Synergistic effect of all the identified compounds was determined via binding energy values computed through docking simulations. Binding orientations showed that all the compounds interact with amino acid residues present in the peripheral anionic site (PAS) and catalytic anionic site (CAS) amino acid residues, oxyanion hole and acyl pocket *via* π-π stacking interactions and hydrogen bond interactions.

**Supplementary Information:**

The online version contains supplementary material available at 10.1186/s12906-021-03420-0.

## Background

Functional food is a term used for the processed food ingredients that exhibit or regulates various physiological functions beside nutritional values. The word was introduced in Japan in the mid-1980s and use of functional foods is regulated by Japanese Ministry of Health and Welfare under the name Foods for Specified Health Use (FOSHU) [1]. More than hundred food products have been recognized by FOSHU, yet this term is relatively new in other countries. According to reports, the market value of functional foods is about 28.9 billion US dollars, though it greatly varies is different countries [2]. But it believed that functional foods would play a significant role in the prevention and treatment of diseases and promotion of health at affordable cost. The role of natural products-based diets in the prevention and treatment of diseases is evident from various studies [3, 4]. A study revealed that the risk of cancer development is less among fruits and vegetables consumers when compared with non-users [5]. It is now well understood that secondary metabolites in these functional foods are responsible for various pharmacological properties including prevention of diseases and other pathological targets [5, 6].

In the modern era, numerous health problems are known to be cured by modulation of nutritional habits and use of edible natural products in the diet [7, 8]. Natural products have been used in various types of food items for therapeutic effects and preservation [9–11]. Among these the nutrition, essential oils and a variety of different plants extracts, and their spices have been a huge attention owing to their safety and efficacy profiles [12–14]. Literature showed that variety of herbs, spices and their essential oils get from medicinal plants are thoroughly employed for the treatment of neurodegenerative diseases, growing and survival of neuronal cell, physical and mental performance on experimental bases in which most of them are scientifically proved [15, 16]. Cinnamon as well as cumin has a verity of anti-microbial, and antioxidants components [17]. Cumin and their chemical constituents use as anti-inflammatory and analgesic agents [18]. Cinnamon extract has been found to inhibit tau aggregation, an important hallmark of Alzheimer's disease (AD) [19]. Ginger used as food ingredient, is effective in inflammation [20], cough, asthma, muscle pain, bleeding, nausea [21]. Ginger in addition to other therapies has been recommended for brain disorders including paralysis via ischemic stroke [22]. Crude extract of ginger were previously reported to modulate brain cholinesterase's and reduce amyloid load in brain tissues [23].

Alzheimer’s disease (AD) is a progressive neurodegenerative disorder which is characterized by cognitive dysfunction, behavioral turbulence and imperfection in activities of daily life [24]. The cognitive defect in AD is due to deterioration of cholinergic neurons particularly at the basal forebrain leading to impairment in neurotransmission in cerebral cortex and other brain parts [25]. An important target in AD is the use of cholinesterase inhibitors to overcome deficiency of a vital neurotransmitter acetylcholine (ACh) [26–28]. Use of free radicals scavengers are also indicated in several diseases including AD [29, 30]. In AD patients Aβ cause liberation of excessive free radicals thus causing neurodegeneration and leading to chronic diseases like AD [31]. Antioxidants attenuate inflammation pathway by scavenging free radicals and ROS. So, the antioxidants may be useful in the protection from neurodegeneration in AD [32, 33]. This study was designed to assess the neuroprotective role of three important edible oils in the context of their enzyme inhibitory and antioxidant potentials.

## Methods

### Oil analysis

Fresh rhizome of ginger and seeds of cumin and cinnamon were purchased from the market at Chakdara, Pakistan and subjected to hydro-distillation using a Clevenger apparatus coupled with condenser [34]. Distillation was continued at 100°C till enough oils were collected. Thereafter, anhydrous sodium sulfate was used for removal of traces of water and were refrigerated at -30°C till further use.

### GC–MS analysis

For the GC–MS analyses ion trap MS spectrometer and a DB-1 MS fused silica nonpolar capillary column (30 m length, with 0.25 mm internal diameter, and 0.25 μm film thickness) was used with helium as carrier gas. The oven temperature was held for 5 min at 50 ^o^C, then increased from 50 to 250 ^o^C at 4 ^o^C per min and held isothermal for 10 minutes. The Injector and MS transfer line temperatures were set at 250 and 290^o^C, respectively. The Ion source temperature was 200 ^o^C while the volume injection was 1 μl. The sample component ionization was performed by EI-MS (70 eV). The Mass range was from m/z 28 to 650 amu. Scan time was 0.5 s with 0.1 s inter scan delay. Identification and quantification of the essential oils components was performed on the basis of GC retention indices and computer matching with the NIST- 2005, Wiley 275 and TRLIB Library, further uncertain identification was made by the comparison of the fragmentation patterns of mass spectra with previously reported in the literature [35, 36].

### DPPH anti-radicals study

The DPPH free radicals scavenging capacity of our oils was analyzed following standard protocol [37]. Briefly, a 0.004% methanolic solution of DPPH (200 μL) was mixed with equal volume of previously prepared samples solutions (1000, 500, 250, 125, 62.5, 31.25 μg/ml). The resultant mixture was maintained at room temperature for about 30 min. Pure methanol and Ascorbic acid solutions were used as negative and reference agents respectively. Absorbance's were measured at 517 nm via UV spectrophotometer and radicals scavenging was calculated as;

$$\mathrm{Percent}\kern0.5em \mathrm{scavenging}\kern0.5em =\frac{\mathrm{Absorbance}\kern0.5em \mathrm{of}\kern0.5em \mathrm{control}-\mathrm{Absorbance}\kern0.5em \mathrm{of}\kern0.5em \mathrm{test}\kern0.5em \mathrm{samples}}{\mathrm{Absorbance}\kern0.5em \mathrm{of}\ \mathrm{control}}\times 100$$

### ABTS anti-radicals study

The anti-radicals potentials of selected oils was further evaluated via another in-vitro assay following our reported protocol [38]. In brief, 100 ml of 7 mmol ABTS methanolic solution was added to 100 ml of 2.45 mmol KH_2_PO_4_ solution followed by addition of 100 ml water. The resultant mixture as overnight incubated at dark to generate sufficient free radicals. Subsequently, ABTS solution absorbance was adjusted to 0.7 at 745 nm via addition of 50% methanolic solution. Thereafter, 3ml of ABTS solution was added to 3 ml of samples in UV covet and absorbance was recorded via UV spectrophotometer. Ascorbic acid was used as control drug in the study. ABTS scavenging activity was calculated as;

$$\mathrm{Percent}\kern0.5em \mathrm{scavinging}\kern0.5em =\frac{\mathrm{Absorbance}\kern0.5em \mathrm{of}\kern0.5em \mathrm{Control}-\mathrm{Absorbance}\kern0.5em \mathrm{of}\ \mathrm{sample}}{\mathrm{Absorbance}\kern0.5em \mathrm{of}\ \mathrm{Control}}\times 100$$

### Cholinesterase inhibition assays

Anti-cholinesterase potentials of selected essentials oils were evaluated by some modification of the spectrophotometric analysis respectively followed by [39]. Different dilutions of plant fraction were made ranging from (1000 to 62.5 μg/ml). Briefly, 10 μl each of acetylcholinesterase and butyrylcholinesterase 0.03 U/ml solution were mixed with 410 μL of different essential oils samples taken in a cuvette. Subsequently, DTNB solution (10 μL) was transferred to the mixture solution, mixed well and incubated at 30°C for 15 min. To start enzymatic reaction, 10μL substrates were added to the reaction mixture. Absorbance's were recorded at 412 nm via microplate reader.

Percent inhibition of AChE and BChE were evaluated by judgment of rates of reaction of tests samples comparative to that of control. Reaction mixture containing all substances without tested sample was used as control. All experiments were repeated three times. Galantamine, the anti-cholinesterase kind of drug was used as standard. The percent inhibition and percent enzymatic potential were calculated as follows;

$${\displaystyle \begin{array}{c}\mathrm{V}=\frac{\Delta \mathrm{Abs}}{\Delta \mathrm{t}}\\ {}\%\kern0.5em \mathrm{enzyme}\kern0.5em \mathrm{activity}=\frac{\mathrm{v}}{\mathrm{v}\max}\times 100\\ {}\%\kern0.5em \mathrm{enzyme}\kern0.5em \mathrm{activity}=100-\%\kern0.5em \mathrm{enzyme}\kern0.5em \mathrm{activity}\end{array}}$$

### Determination of IC_50_

From inhibition values IC_50_ were determined using their dose-response curves [40].

### Estimation of kinetic parameters

The Kinetic parameters were estimated via construction of Lineweaver-Burk plots (1/*v* versus 1/[*s*]) where *v* is apparent velocity reaction and [*s*] at the concentrations used for substrates and enzymes. The V_max_ and K_m_ values were determined via Michaelis Menten kinetics [41].

### Docking studies

Identified compounds were docked against the target site of enzymes using Molecular Operating Environment (MOE 2016.0802) suite [41, 42]. Briefly, crystal structure of Torpedo California AChE (TcAChE, PDB code 1EVE) with native ligand donepezil was acquired from Protein Data Bank. Whereas, legands preparation, identification of enzymes active sites as well as detailed docking method was adopted from our previously reported procedure [43, 44]. Likewise, three-dimensional interaction plot of the drug-enzyme complex was analyzed by using Discovery Studio Visualizer (Dassault Systems BIOVIA 2017) [45].

### Statistical analysis

Data were expressed as mean ± S.E.M. Multiple group means of parametric data sets were compared using two-way analysis of variance (ANOVA) after it was determined that the data conformed to a normal distribution. If an overall significance was found, a multiple-comparisons test was used using GraphPad Prism 5 (GraphPad Software Inc. San Diego CA, USA) [46]. A value of *P* < 0.05 was considered as significant.

## Results

### Phytochemistry of oils

**Fig. 1:**
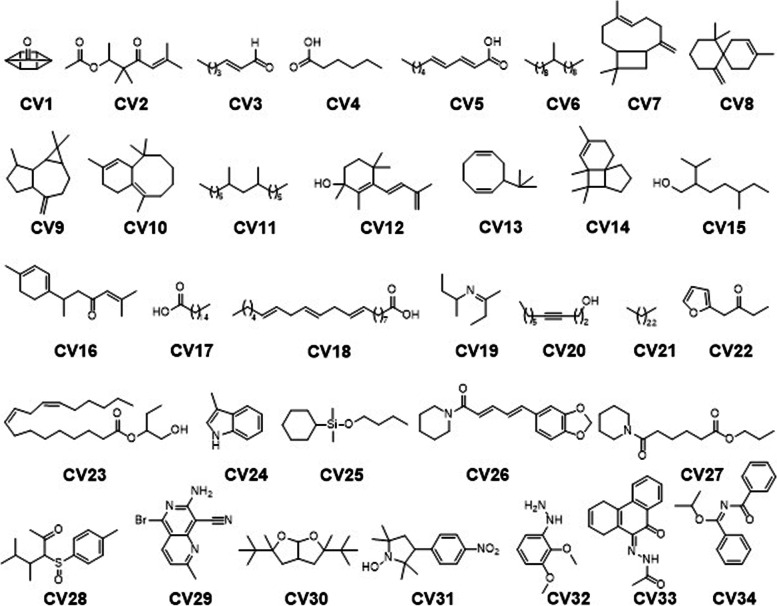
Identified compounds in the oils of *Cinnamomum verum*

**Fig. 2:**
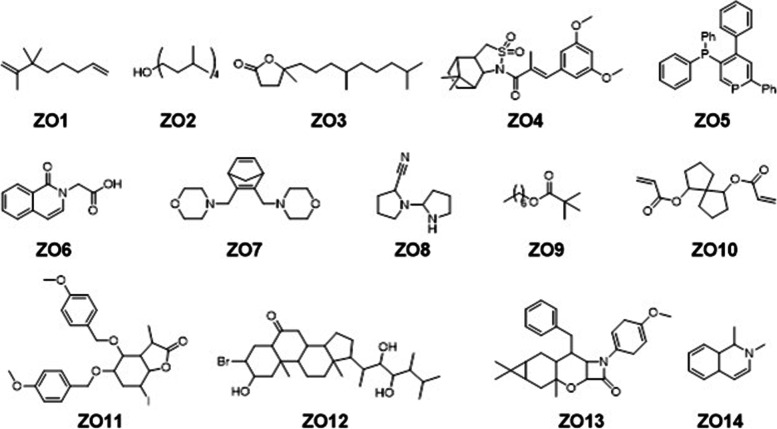
Identified compounds in the oils of *Zingiber officinale*

**Fig. 3:**
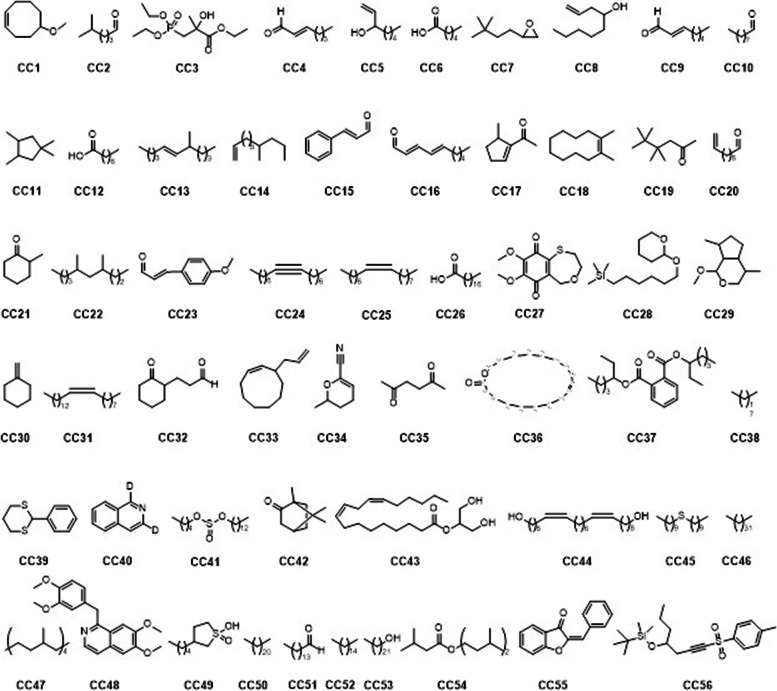
Identified compounds in the oils of *Cuminum cyminum*

GC-MS analysis of the selected oils revealed the presence of potent secondary metabolites. We identified thirty-four (34) compounds in the oils obtained from cinnamon as shown in Fig. 1 (Compound CV1 – 34). The GCMS details for the identified compounds are provided in Table S1 of the supporting information. The GCMS analysis of *Zingiber officinale* oil confirm the presence of lesser number of compounds (Compounds ZO1 – 14, Fig. 2). Total of fourteen potent compounds were identified. The chemical names, retention times, molecular formula and other details of the identified compounds are shown in Table S2 of the supporting information. The oils obtained from *Cuminum cyminum* showed relatively larger number of bioactive phytochemicals. In *C. cyminum* oil, total of fifty-six compounds were identified by GCMS analysis. The structures of compounds (CC1 – 56) are shown in Fig. 3 while the chemical names, retention times with the specified method and other details are provided in Table S3 of the supporting information file.

### Antioxidant studies

**Fig. 4:**
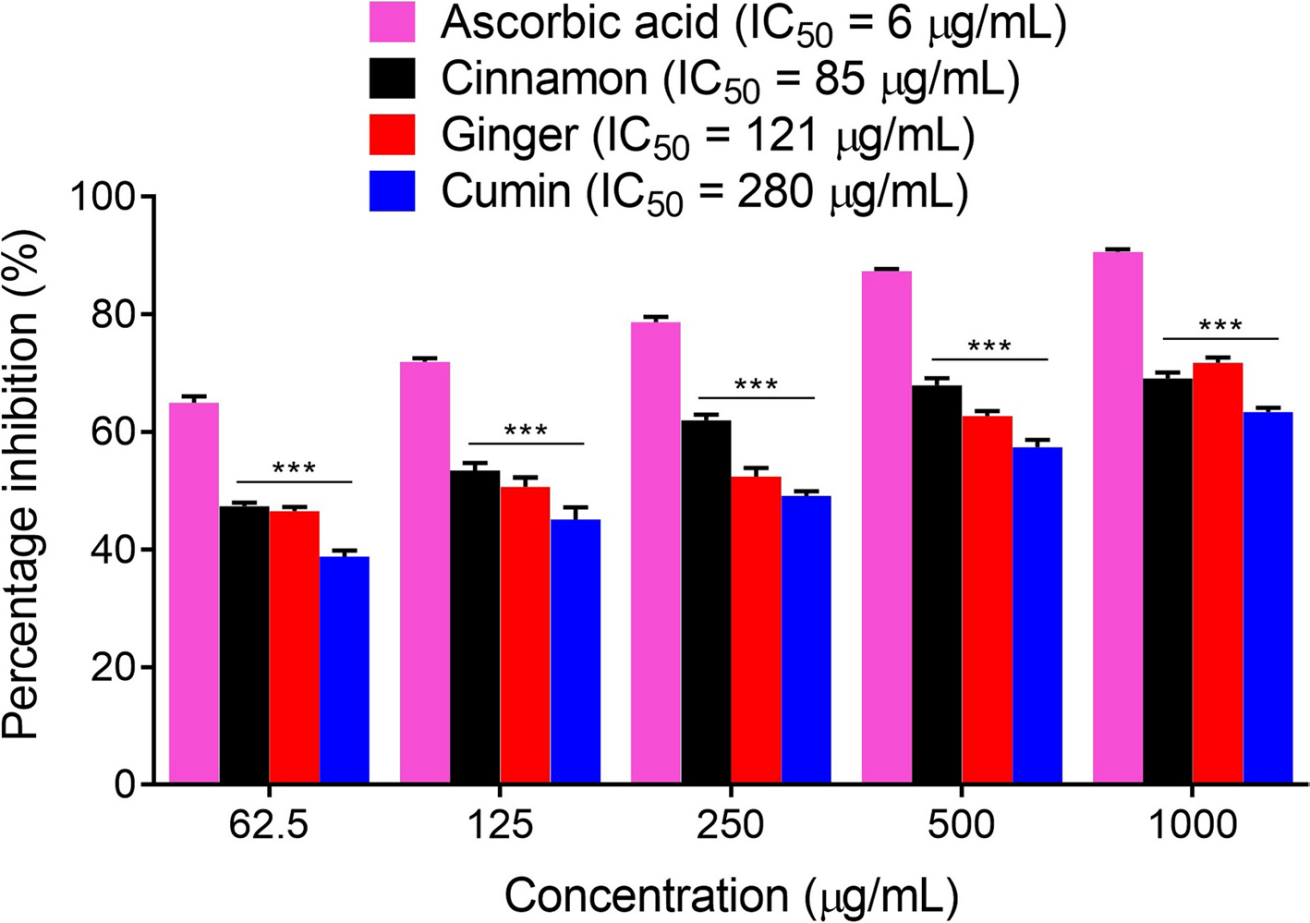
The percentage inhibition and the IC_50_ values of cinnamon, ginger, cumin, and the positive control ascorbic acid in the DPPH antioxidant assay. ^***^P < 0.001 as compared to ascorbic acid, two-way repeated measures ANOVA followed by *post hoc* Bonferroni’s analysis. Data are presented as mean percentage inhibition ± SEM of three different experiments

**Fig. 5:**
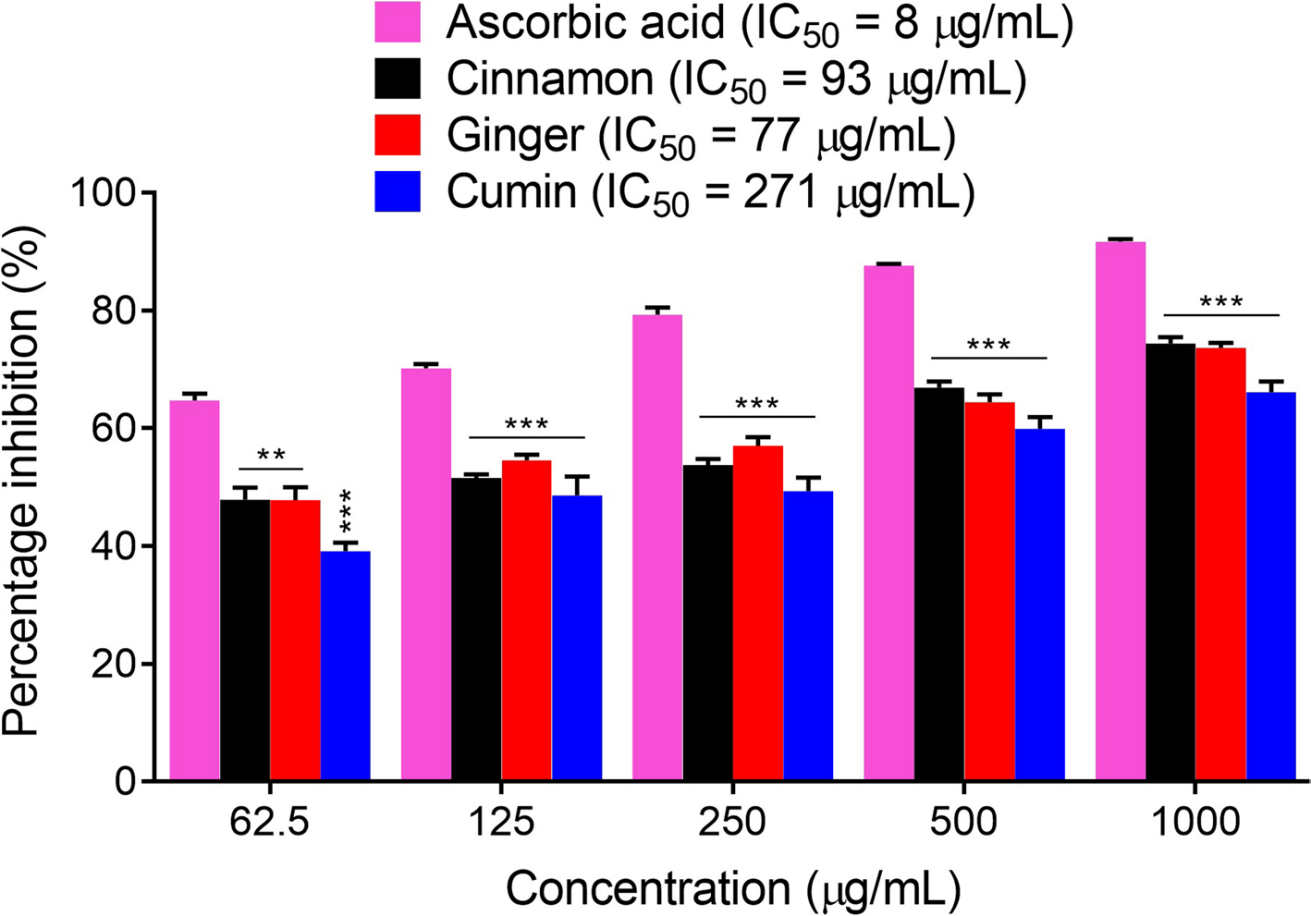
The percentage inhibition and the IC_50_ values of cinnamon, ginger, cumin, and the positive control ascorbic acid in the ABTS antioxidant assay. ^**^P < 0.01, ^***^P < 0.001 as compared to ascorbic acid, two-way repeated measures ANOVA followed by *post hoc* Bonferroni’s analysis. Data are presented as mean percentage inhibition ± SEM of three different experiments

The three edible oils, i.e., cinnamon, ginger and cumin were tested for their antioxidant assays using DPPH (Fig. 4) and ABTS (Fig. 5) free radicals scavenging assays. All the three oils were tested at concentrations of 62.50-1000 μg/ml in comparison to the positive control, ascorbic acid in both DPPH and ABTS assays as shown in Table S4 of the supporting information. The three edible oils have strong DPPH free radicals scavenging tendency and in comparison to the positive control, ascorbic acid, these oils showed a significant difference in their percentage inhibitions at the tested concentrations of 62.50-1000 μg/ml (*P* < 0.001). In the DPPH assay, the calculated values of IC_50_ were 85, 121 and 280 μg/ml for cinnamon, ginger and cumin oils respectively, while for the positive control, ascorbic acid it was observed as 06 μg/ml. Similarly, in the ABTS assay, the three edible oils showed marked antioxidant propensity as observed from the percentage inhibition of ABTS free radicals, which were found to be significantly different at the tested concentrations of 62.5 μg/ml (*P* < 0.01 for cinnamon and ginger, *P* < 0.001 for cumin), and 125-1000 μg/ml (*P* < 0.001) as compared to the positive control, ascorbic acid. At highest tested concentration, the cinnamon, ginger and cumin exhibited 74.40, 73.65 and 66.17% ABTS free radicals scavenging respectively, while the positive control ascorbic acid exhibited 91.69% ABTS free radicals scavenging. In the ABTS assay, the calculated IC_50_ values were 93, 77, 271 and 08 μg/ml for cinnamon, ginger, cumin and ascorbic acid respectively.

### Cholinesterase inhibition assays

Selected samples were tested for AChE/BChE inhibitions as shown in Table 1. In both assays, 1000, 500, 250, 125 and 62.50 μg/ml concentrations were used. The cinnamon oil showed considerable AChE inhibitory assay among the tested samples. Cinnamon oil exhibited 78.33, 71.33, 63.94, 61.90 and 59.08% inhibitions at concentration of 1000, 500, 250, 125 and 62.50 μg/ml respectively giving IC_50_ value of 21 μg/ml. In comparison the observed IC_50_ of the galantamine was 08 μg/ml. In the same assay, ginger and cumin oils gave IC_50_ values of 88 and 198 μg/ml respectively. Similarly, in BChE assay, cinnamon, ginger and cumin oils exhibited IC_50_ values of 106, 101 and 37 μg/ml respectively as shown in Table 1.

**Table 1:** Results of cholinesterase inhibitory potentials of different food oils

Samples	Concentration (μg/mL)	Percent inhibition (AChE)	AChE IC_50_ (μg/mL)	Percent inhibition BChE	BChE IC_50_ (μg/mL)
Cinnamon	1000 500 250 125 62.5	78.33 ± 0.88*** 71.33 ± 1.01*** 63.94 ± 1.41*** 61.90 ± 1.45** 59.08 ± 2.5^ns^	21	68.26 ± 1.19*** 63.32 ± 1.28*** 58.36 ± 2.54*** 52.26 ± 2.60*** 43.33 ± 1.45***	106
Ginger	1000 500 250 125 62.5	71.63 ± 1.68*** 68.40 ± 2.05*** 59.97 ± 0.96*** 53.91 ± 1.06*** 46.97 ± 1.70***	88	74.30 ± 0.79*** 65.79 ± 1.10*** 61.74 ± 1.75*** 56.19 ± 1.46*** 42.36 ± 3.18***	101
Cumin	1000 500 250 125 62.5	70.63 ± 1.45*** 60.70 ± 0.80*** 51.10 ± 2.78*** 48.29 ± 1.21*** 44.59 ± 0.59***	198	86.51 ± 2.57^ns^ 77.38 ± 2.04** 69.93 ± 0.79** 63.24 ± 1.72** 56.90 ± 1.63**	37
Galantamine	1000 500 250 125 62.5	94.08 ± 0.81 90.69 ± 0.42 83.91 ± 1.04 69.63 ± 0.97 64.04 ± 1.39	08	91.62 ± 0.64 86.48 ± 1.24 79.28 ± 1.73 72.90 ± 0.95 66.23 ± 1.21	06

The values are presented as mean ± SEM (*n* = 3). The asterisk shows that the significance levels in comparison with that of the positive control: Data were analyzed via TWO-WAY ANOVA followed by Bonferroni *post hoc* test, ^∗^ *P* < 0.05; ^∗∗^ *P* < 0.01, ^∗∗∗^ *P* < 0.001, *ns; P* > 0.05

### Kinetic studies

**Fig. 6:**
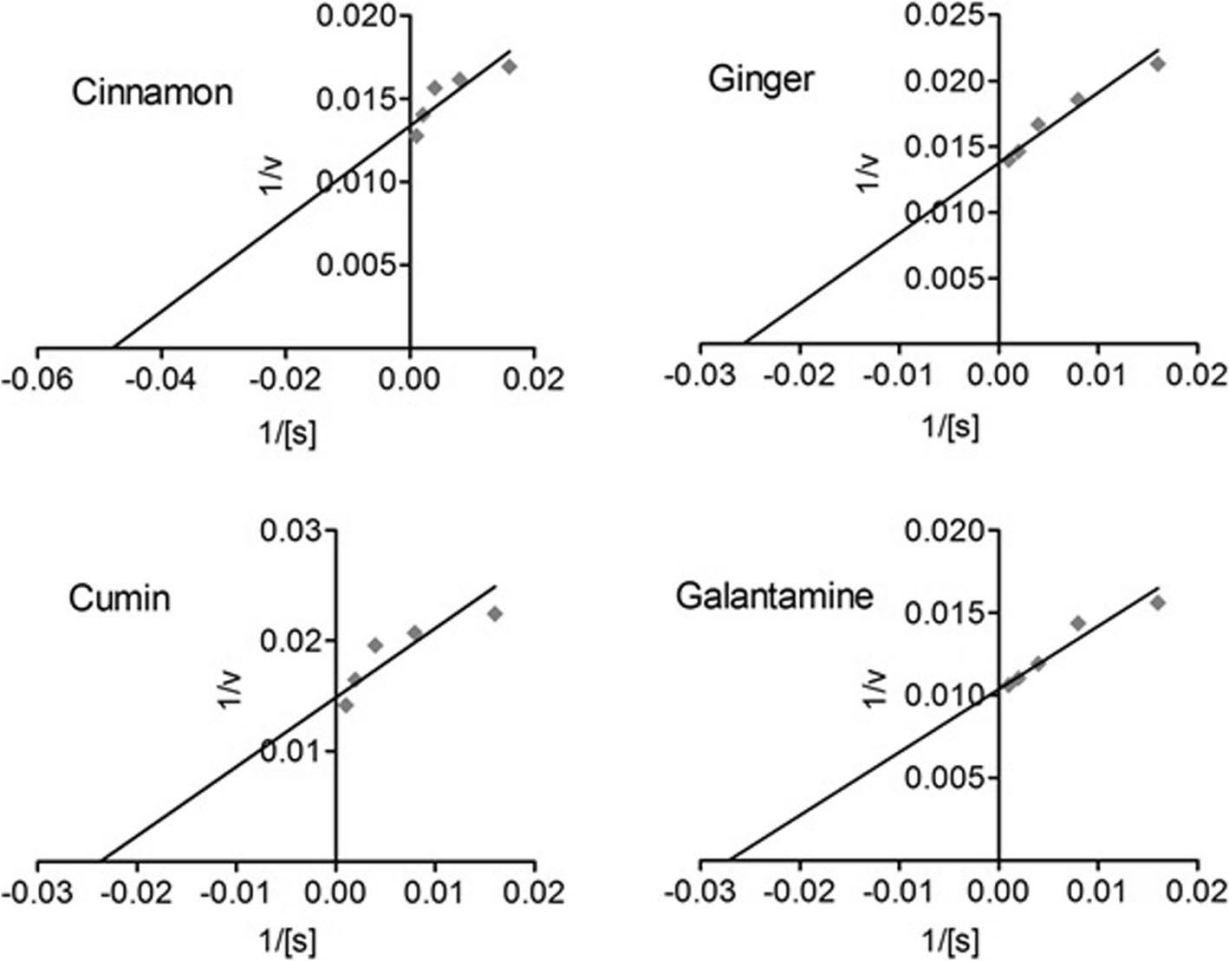
Lineweaver-Burk plots of acetylcholinesterase inhibition

**Fig. 7:**
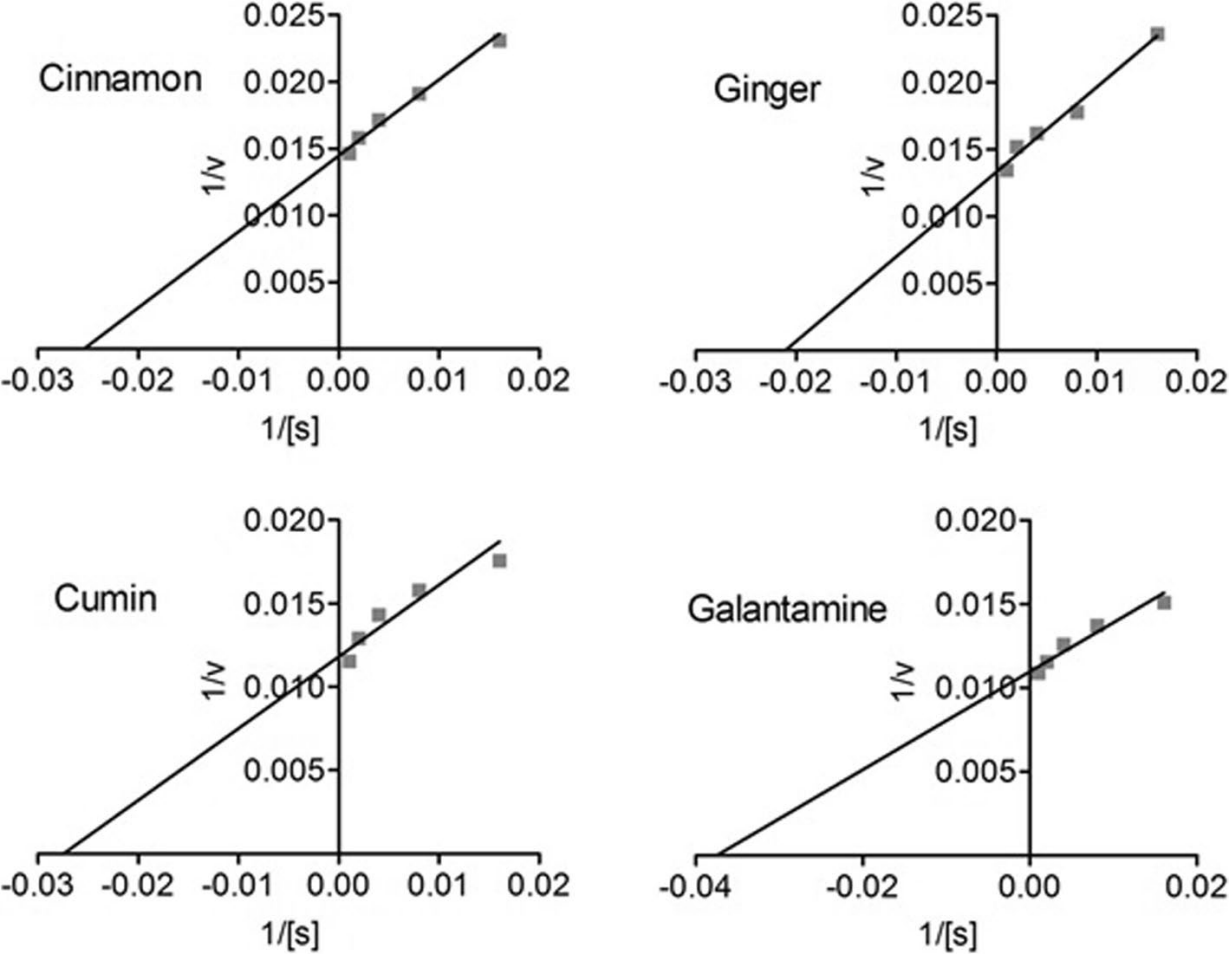
Lineweaver-Burk plots of butylcholinesterase inhibition

In kinetics studies, cinnamon, ginger, and cumin showed strong inhibitory potential against AChE/BChE calculated from the *V*_*max*_ and *K*_*m*_ and inveterate from the Linewear-Burk plots for the respective enzymes (Figs. 6 and 7) [47]. For AChE inhibition, the *V*_*max*_ and *K*_*m*_ values were noted as 74.84 μg/min and 20.87 μg/ml for cinnamon, 72.72 μg/min and 38.88 μg/mL for ginger, and 67.35 μg/min and 42.23 μg/ml for cumin. The positive control, galantamine showed a robust inhibition of AChE having *V*_*max*_ and *K*_*m*_ values of 96.47 μg/min and 36.87 μg/mL, respectively. Similarly, the *V*_*max*_ and *K*_*m*_ values for BChE inhibition also revealed a potent inhibitory potential of cinnamon (69.18 μg/min and 39.43 μg/mL), ginger (75.06 μg/min and 47.48 μg/ml), and cumin (84.70 μg/min and 36.44 μg/ml), respectively. A high-grade inhibitory activity was observed for the positive control, galantamine (91.09 μg/min and 26.69 μg/ml).

### Docking studies against acetylcholinesterase (AChE) target

Binding orientations, interactions and energy data has a key role to explore the possible mechanism of inhibition. In case of AChE inhibition, interactions with the peripheral anionic site (PAS) and catalytic anionic site (CAS) amino acid residues are important for further chemical modification and structure activity relationship exploration. In case of plant extracts, the role of each constituent is also important to determine the synergistic effect. Hence, to further strengthen our study, we carried out docking simulation using MOE software.

**Fig. 8:**
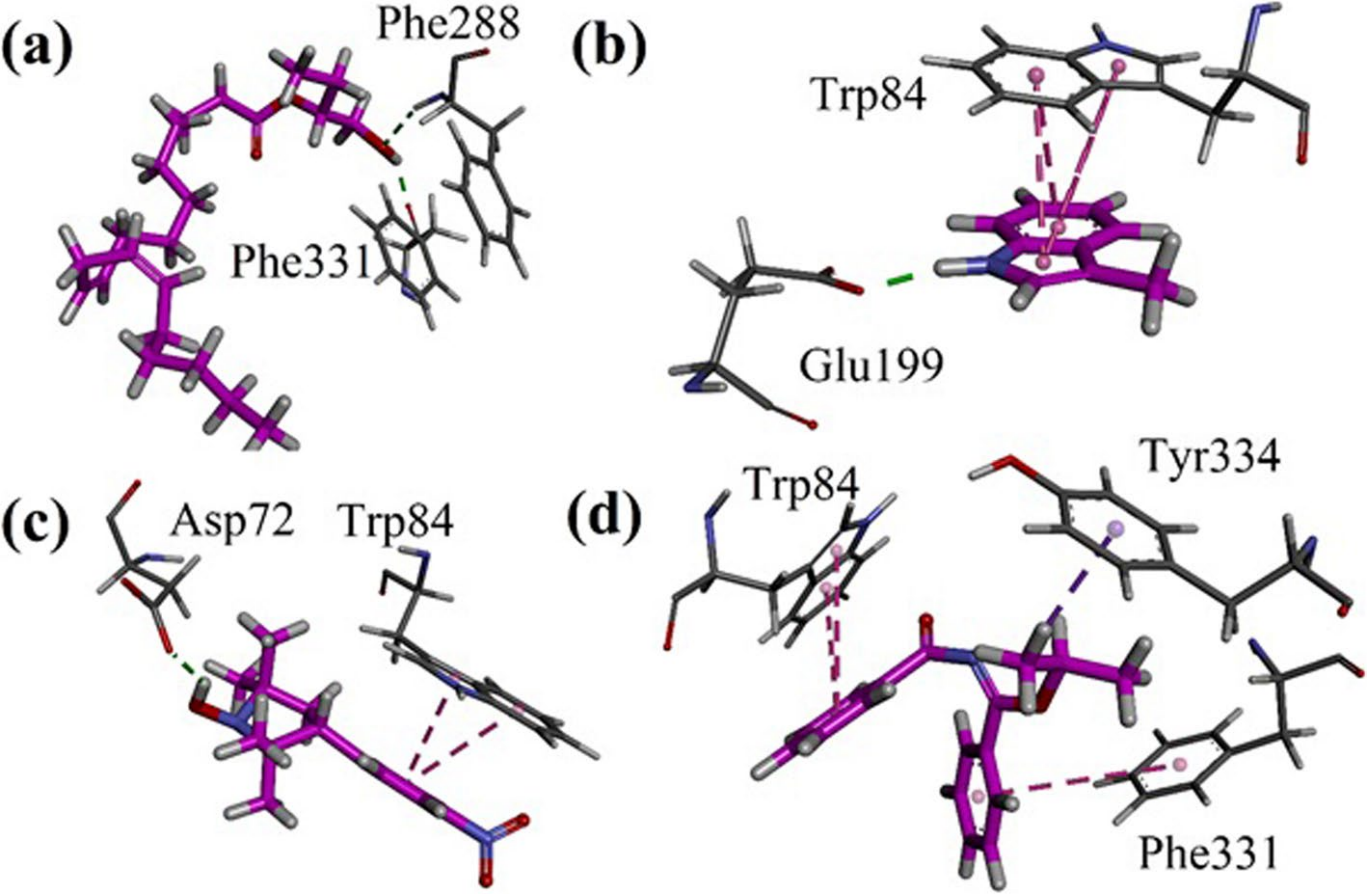
Results of docking studies on compounds identified from *Cinnamomum verum* showing important residues with **a** CV5; **b** CV24; **c** CV31 and **d** CV34 into the binding site of Torpedo californica AChE (*Tc*AChE, PDB code 1EVE)

**Fig. 9:**
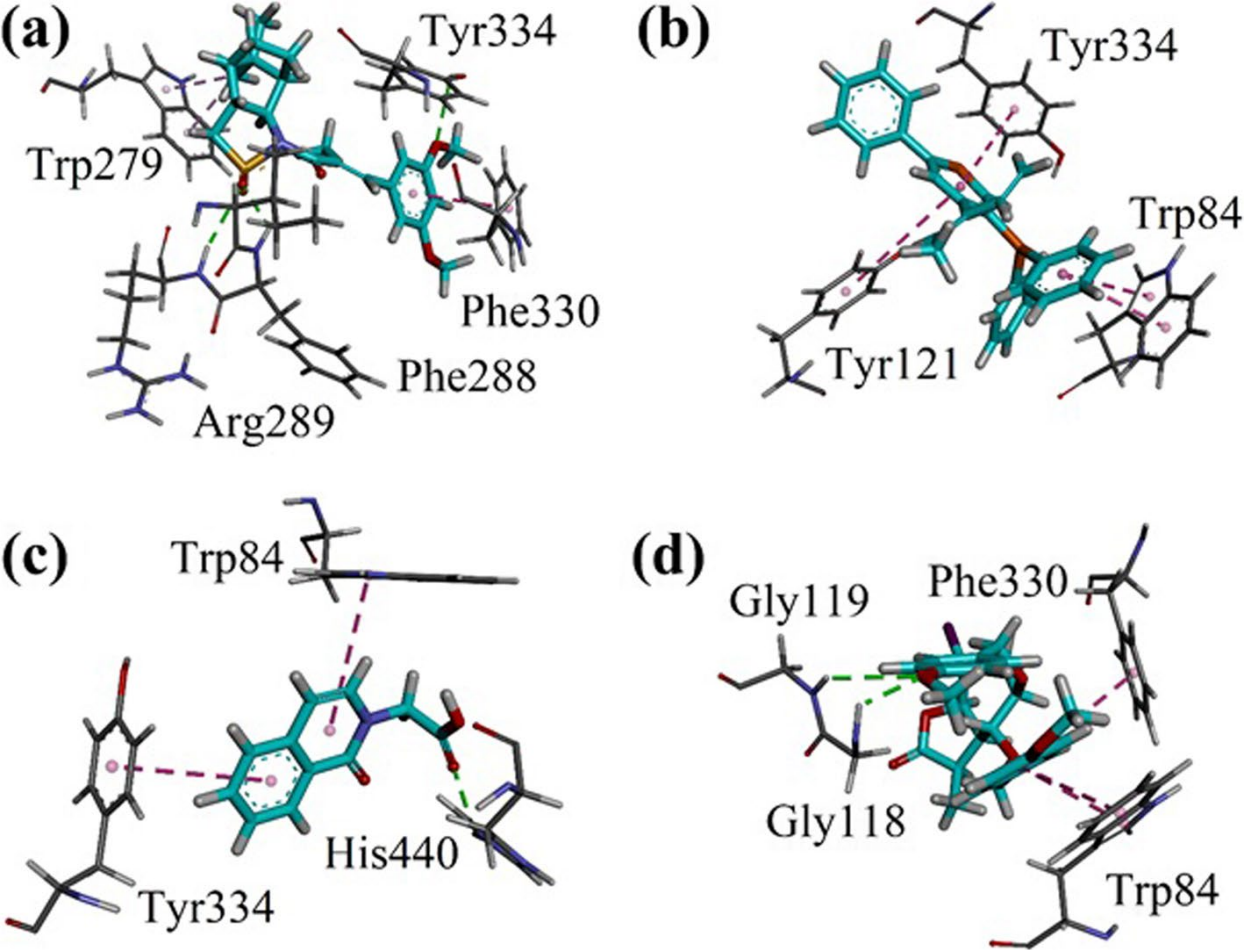
Results of docking studies on the compounds identified from *Zingiber officinale* showing important residues with **a** ZO4; **b** ZO5; **c** ZO6 and **d** ZO11 into the binding site of Torpedo californica AChE (TcAChE, PDB code 1EVE)

**Fig. 10:**
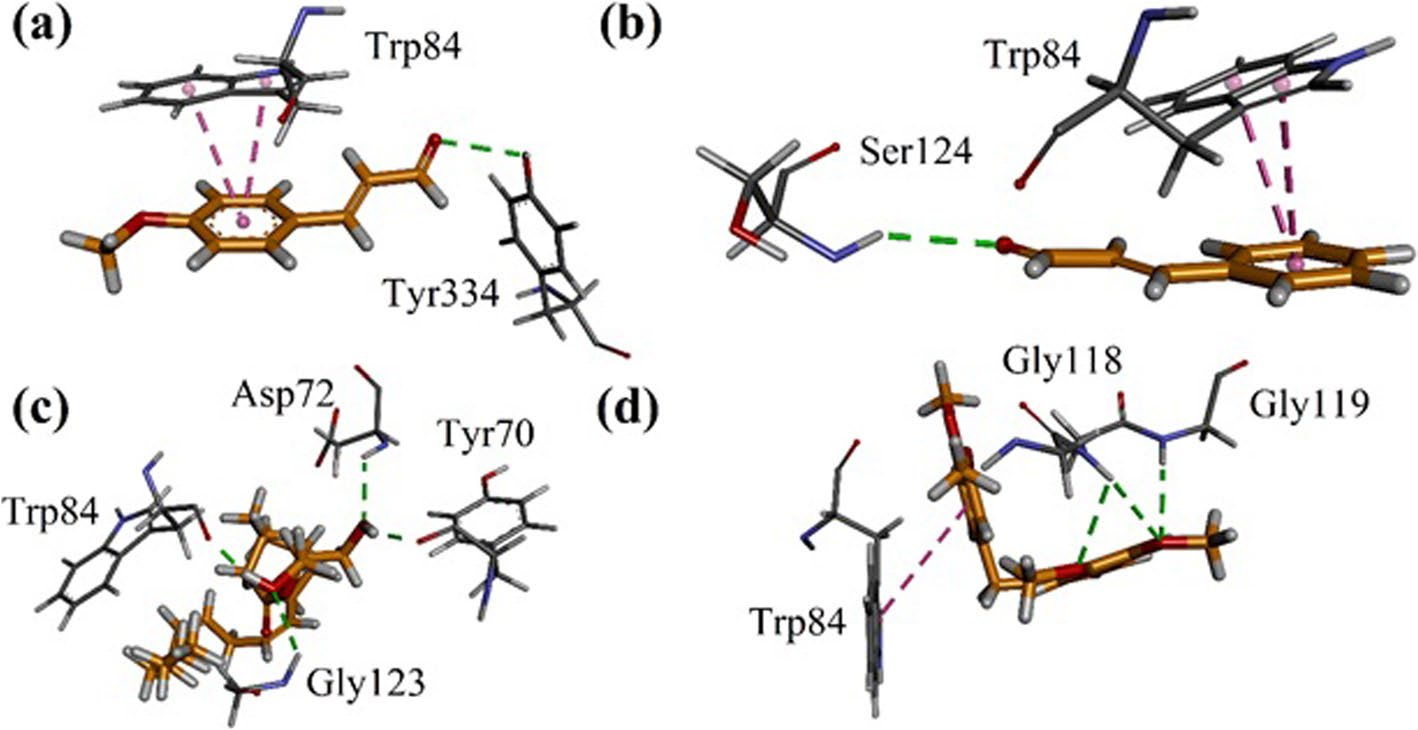
Results of docking studies on the compounds identified from *Cuminum cyminum* showing important residues with **a** CC15; **b** CC23; **c** CC43 and **d** CC48 into the binding site

We docked all the identified compounds from oils of cinnamon, ginger, and Cumin into the binding site of Torpedo California AChE (TcAChE, PDB code 1EVE). Three-dimensional (3-D) interaction plots of important compounds with binding energy value greater than -7.00 kcal/mol are shown in Figs. 8, 9 and 10. All the identified compounds from cinnamon are shown in pink stick form. While compounds from ginger and cumin are shown in turquoise and orange color, respectively. Compounds containing aromatic rings forms π-π stacking interactions with catalytic anionic site residue Trp84, Phe330 and Phe331. Peripheral anionic site (PAS) residues Tyr121 and Tyr334 also formed π-π stacking interactions with the aromatic rings. Hydroxyl, and carbonyl oxygen groups forms hydrogen bond interactions with amino acid residues present in the active of AChE. Compounds with aliphatic chains showed binding energy values between 4.0 and 5.5 kcal/mol.

Interaction plot of the identified compounds from *C. verum* (CV5, CV24, CV31 and CV34) are shown in Fig. 8a-d. Hydroxyl group of CV5 forms hydrogen bond interactions with Phe288 (acyl pocket residue) and Phe331 (Fig. 8a). Indole ring of CV24 forms bifurcated π-π stacking interactions with indole ring of catalytic anionic site residue Trp84. While -NH forms hydrogen bond interactions with Glu199 (Fig. 8b). Compound CV31 and CV35 also forms π-π stacking interactions with Trp84 (Fig. 8c-d).

Interaction plots of compounds ZO4, ZO5, ZO6 and ZO11 identified from *Z. officinale* are shown in Fig. 6a-d. ZO4 forms hydrogen bond interactions with Phe288, Arg289 and Tyr334. Phe330 forms π-π stacking interactions with dimethoxyphenyl ring. PAS residue Trp279 forms π-alkyl interactions (Fig. 9a). Identified compound ZO5 forms π-π stacking interactions with Trp84. While PAS residues Tyr121 and Tyr334 forms π-π T-shaped interactions with ring (Fig. 9b). ZO6 forms π-π T-shaped interactions with Trp84 and Tyr334. While catalytic triad residue His440 forms hydrogen bond interactions with carbonyl oxygen (Fig. 9c). In case of ZO11, Trp84 forms π-π stacking interactions and Phe330 forms π-π T-shaped interactions. Oxyanion hole residues Gly118 and Gly119 establish hydrogen bond interactions with methoxy oxygen atom (Fig. 9d).

Interaction plots of compounds CC15, CC23, CC43 and CC48 identified from *C. cyminum* are shown in Fig. 10a-d. CC15 and CC23 form π-π stacking interactions with Trp84. While hydrogen bond interactions were found with Tyr334 and Ser124 respectively (Fig. 10a-b). Compound CC43 forms four hydrogen bond interactions. Here, Trp84 forms hydrogen bond interaction with hydroxyl group. PAS residues Tyr70 and Asp72 also forms hydrogen bond interactions with hydroxy group (Fig. 10c). Compound CC48 forms π-π stacking interactions with Trp84. Oxyanion hole residues Gly118 and Gly119 establish hydrogen bond interactions with methoxy oxygen atoms (Fig. 10d).

## Discussions

Free radicals generated during metabolic processes are involved in the progression of numerous diseases including AD. The human immune system antioxidant enzymes normally neutralize these free radicals and thus protect us against the hazardous actions of these radicals [48, 49]. However, under unusual circumstance when production of these free radicals is excessive or human immune system is compromised, then these free radicals exhibit diverse degenerative effects [50, 51]. Subsequently, exogenous supplementation of antioxidants is extremely necessary to counteract the unwanted effects of these free radicals. Currently beside new drugs discovery, use of functional foods is gaining attention. These substances beside their nutritional values significantly contribute towards the prevention and treatment of various diseases. The essential oils used in the current study has a wide use in foods and are common spices. Subsequently, they were tested for their in vitro scavenging potentials against free radicals and inhibitory activities against vital enzymes implicated in AD. In the current study, we employed two extensively used antioxidant methods including DPPH and ABTS assays [52, 53]. In DPPH radical scavenging assay, cinnamon, ginger, cumin and standard exhibited IC_50_ values of 85, 121, 280 and 06 μg/ml respectively. The IC_50_ value of cinnamon oil was very comparative to the standard drug ascorbic acid (Fig. 4). These results suggest that cinnamon essential oil has high radical scavenging activity. Further, ABTS assays was also used to evaluate the antioxidant potentials of selected samples. When ABTS radical is mixed with an antioxidant agent, ABTS radical accept electron and is converted to a non-radical form. Color change indicates reduction of the ABTS radical [54]. Cinnamon, ginger and cumin oils showed considerable ABTS radicals scavenging activity. The IC_50_ values of cinnamon, ginger and cumin and standard drug were 93, 77, 271 and 08 respectively (Fig. 5). The details about DPPH and ABTS assays are provided in Table S4 of the supporting information.

Cholinesterase’s are important enzymes responsible for removal of ACh from the synaptic cleft. The enzyme metabolizes acetylcholine after their interaction with cholinergic receptors and is recycled [55]. As there is deficiency of ACh in AD patients, so an important therapeutic option is to use inhibitors of cholinesterase's so that any acetylcholine available at synapses may remain for prolong time and symptoms related to acetylcholine deficiency will be relived [56]. Based on the idea, few cholinesterase inhibitors were developed and approved for clinical use. Among these, one drug (galantamine) is from natural sources whereas, rivastigmine is synthetic derivative of a naturally isolated compounds. However, these agents have limited efficacy and some side effects. So, there is dire need for the discovery and development of more useful cholinesterase potentially from natural sources. In the current study, our test sample exhibited considerable cholinesterase inhibitory potentials. The IC_50_ values of cinnamon, ginger, cumin and standard drug against acetyl cholinesterase were 21, 88, 198 and 08 μg/ml respectively. Whereas the IC_50_ values of cinnamon, ginger, cumin standard against BChE enzyme were 106, 101, 37 and 06 μg/ml respectively (Table 1).

In cinnamon oil several compounds were identified which are previously reported for diverse antioxidant and cholinesterase inhibitory potentials. For instance, heptenal present in *Cardiospermum halicacabum* is reported to possess antioxidant and anti-cholinesterase potentials [57]. Likewise, caproic acid is reported both for cholinesterase [58] and antioxidant properties [59]. Phytochemicals including 2,4-Decadien-1-al, 10-Methylnonadecane, Trans-Caryophyllene, BETA-Chamigrene, [60–66]. Numerous compounds identified in cinnamon oil including -(-) Alloaromadendren, Beta-Himachalene and Italicene exhibit free radicals scavenging and anti-cholinesterase potentials [67–69]. Likewise, in ginger oil several bioactive compounds are previously reported. For instance, 1-Hexadecanol, 3,7,11,15-tetramethyl-, Pivalate and Isoquinonline are previously reported known anti-oxidant and enzyme inhibiting metabolites [70–72]. Cumin oil is also enriched with neuroprotective metabolites. Among the identified compounds in cumin oil, 1-Nonen-4-ol, E-2-octenal, n-Nonylaldehyde and several other metabolites are reported to possess both radicals scavenging and cholinesterase inhibitory potentials [73–75]. The overall neuroprotective potentials of the oils might be attributed to the presence of these bioactive secondary metabolites.

## Conclusions

In the present study cinnamon, ginger and cumin exhibited considerable anti-radical and cholinesterase inhibitory potentials. GCMS revealed the presence of several bioactive compounds which might be implicated in the pharmacological properties of essential oils. Being commonly used spices, these agents might be useful for the prevention of memory-related degenerative disorders like AD. However, furthermore detailed animal studies are required regarding the molecular mechanism and potential use of these agents for the said properties. We docked all the identified compounds from oils of cinnamon, ginger, and Cumin into the binding site of enzyme. Synergistic effect of all the identified compounds was determined *via* binding energy values computed through docking simulations. A number of compounds exhibited binding energy values between 7.0 to 9.5 kcal/mol. Furthermore, interactions with the PAS residues may prevent the AChE induced Aβ-aggregation. Hence, we may conclude here that the identified compounds may also prevent Aβ-aggregation and thus have a role to prevent Alzheimer’s disease.

## Supplementary Information

**Additional file 1: Table S1.** Details of identified compounds in GC-MS analysis of cinnamon oil. **Table S2.** Chemical composition of ginger essential oil. **Table S3.** Chemical composition of cumin essential oil. **Table S4.** Antioxidant of the selected essential oils using ascorbic acid as standard.

## Data Availability

The data is available on request from corresponding authors.

## Abbreviations

CV: Cinnamomum verum
ZO: Zingiber officinale
CC: Cuminum cyminum
GC-MS: Gas Chromatography – Mass Spectrometry
AChE: Acetylcholinesterase
BChE: Butyrylcholinesterase
MOE: Molecular Operating Environment
Cv.Eo: Cinnamon oil
Zo.Eo: Ginger oil
Cc.Eo: Cumin oil
PAS: Peripheral Anionic site
CAS: Catalytic Anionic site
AD: Alzheimer’s Disease
ROS: Reactive Oxygen Species
DPPH: 2,2-diphenyl-1-picrylhydrazyl
ABTS: 2,2'-azino-bis(3-ethylbenzothiazoline-6-sulfonic acid)
PDB: Protein Data Bank

## References

[1] Arai S (1996). Studies on functional foods in Japan—state of the art. Biosci Biotechnol Biochem.

[2] Waltham M. Roadmaps to market: commercializing functional foods and nutraceuticals, decision resources. In.: Inc; 1998.

[3] Ayaz M, Sadiq A, Junaid M, Ullah F, Subhan F, Ahmed J (2017). Neuroprotective and anti-aging potentials of essential oils from aromatic and medicinal plants. Front Aging Neurosci.

[4] Ayaz M, Subhan F, Sadiq A, Ullah F, Ahmed J, Sewell RDE (2017). Cellular efflux transporters and the potential role of natural products in combating efflux mediated drug resistance. Front Biosci.

[5] Block G, Patterson B, Subar A (1992). Fruit, vegetables, and cancer prevention: a review of the epidemiological evidence. Nutr Cancer.

[6] Ayaz M, Junaid M, Ullah F, Sadiq A, Subhan F, Khan MA, Ahmad W, Ali G, Imran M, Ahmad S (2016). Molecularly characterized solvent extracts and saponins from Polygonum hydropiper L. show high anti-angiogenic, anti-tumor, brine shrimp, and fibroblast NIH/3T3 cell line cytotoxicity. Front Pharmacol.

[7] Shah SM, Ullah F, Ayaz M, Sadiq A, Hussain S, Shah SAA, Nadhman A (2019). β-Sitosterol from Ifloga spicata (Forssk.) Sch. Bip. as potential anti-leishmanial agent against leishmania tropica: docking and molecular insights. Steroids.

[8] Chung H-Y, Park YK (2017). Rationale, feasibility and acceptability of ketogenic diet for cancer treatment. J Cancer Prev.

[9] Sadiq A, Ahmad S, Ali R, Ahmad F, Ahmad S, Zeb A, Ayaz M, Ullah F, Siddique AN (2016). Antibacterial and antifungal potentials of the solvents extracts from Eryngium caeruleum, Notholirion thomsonianum and Allium consanguineum. BMC Complement Altern Med.

[10] Akbar S, Subhan F, Shahid M, Wadood A, Shahbaz N, Farooq U, Ayaz M, Raziq N (2020). 6-Methoxyflavanone abates cisplatin-induced neuropathic pain apropos anti-inflammatory mechanisms: a behavioral and molecular simulation study. Eur J Pharmacol.

[11] Ahmad S, Zeb A, Ayaz M, Murkovic M (2020). Characterization of phenolic compounds using UPLC–HRMS and HPLC–DAD and anti-cholinesterase and anti-oxidant activities of Trifolium repens L. leaves. Eur Food Res Technol.

[12] Shah SMM, Ullah F, Shah SMH, Zahoor M, Sadiq A (2012). Analysis of chemical constituents and antinociceptive potential of essential oil of Teucrium Stocksianum bioss collected from the North West of Pakistan. BMC Complement Altern Med.

[13] Aslam H, Khan A-U, Naureen H, Ali F, Ullah F, Sadiq A (2018). Potential application of Conyza canadensis (L) Cronquist in the management of diabetes: in vitro and in vivo evaluation. Trop J Pharm Res.

[14] Saleem U, Khalid S, Zaib S, Anwar F, Ahmad B, Ullah I, Zeb A, Ayaz M (2020). Phytochemical analysis and wound healing studies on ethnomedicinally important plant Malva neglecta Wallr. J Ethnopharmacol.

[15] Kannappan R, Gupta SC, Kim JH, Reuter S, Aggarwal BB (2011). Neuroprotection by spice-derived nutraceuticals: you are what you eat!. Mol Neurobiol.

[16] Ayaz M, Ullah F, Sadiq A, Kim MO, Ali T (2019). Natural products-based drugs: potential therapeutics against Alzheimer’s disease and other neurological disorders. Front Pharmacol.

[17] Ranasinghe P, Pigera S, Premakumara GS, Galappaththy P, Constantine GR, Katulanda P (2013). Medicinal properties of ‘true’cinnamon (Cinnamomum zeylanicum): a systematic review. BMC Complement Altern Med.

[18] Mnif S, Aifa S (2015). Cumin (Cuminum cyminum L.) from traditional uses to potential biomedical applications. Chem Biodivers.

[19] Peterson DW, George RC, Scaramozzino F, LaPointe NE, Anderson RA, Graves DJ, Lew J (2009). Cinnamon extract inhibits tau aggregation associated with Alzheimer's disease in vitro. J Alzheimers Dis.

[20] Grzanna R, Lindmark L, Frondoza CG (2005). Ginger—an herbal medicinal product with broad anti-inflammatory actions. J Med Food.

[21] Mascolo N, Jain R, Jain S, Capasso F (1989). Ethnopharmacologic investigation of ginger (Zingiber officinale). J Ethnopharmacol.

[22] Xutian S, Tai S, Yuan C-S (2014). Handbook of traditional chinese medicine (In 3 volumes).

[23] Ahmed H, Zaazaa AM, Abd El-Motelp B (2014). Zingiber officinale and Alzheimer’s disease: evidences and mechanisms. Int J Pharm Sci Rev Res.

[24] Ayaz M, Junaid M, Ullah F, Subhan F, Sadiq A, Ali G, Ovais M, Shahid M, Ahmad A, Wadood A (2017). Anti-Alzheimer’s Studies on β-Sitosterol Isolated from Polygonum hydropiper L. Front Pharmacol.

[25] Zafar R, Zubair M, Ali S, Shahid K, Waseem W, Naureen H, et al. Zinc metal carboxylates as potential anti-Alzheimer’s candidate: in vitro anticholinesterase, antioxidant and molecular docking studies. J Biomol Struct Dyn. 2020:1–11. 10.1080/07391102.2020.1724569 32013770

[26] Khalil AT, Ayaz M, Ovais M, Wadood A, Ali M, Shinwari ZK, Maaza M (2018). In vitro cholinesterase enzymes inhibitory potential and in silico molecular docking studies of biogenic metal oxides nanoparticles. Inorg Nano-Met Chem.

[27] Ayaz M, Ovais M, Ahmad I, Sadiq A, Khalil AT, Ullah F (2020). Biosynthesized metal nanoparticles as potential Alzheimer’s disease therapeutics. Metal nanoparticles for drug delivery and diagnostic applications.

[28] Ovais M, Ayaz M, Khalil AT, Shah SA, Jan MS, Raza A, Shahid M, Shinwari ZK (2018). HPLC-DAD finger printing, antioxidant, cholinesterase, and α-glucosidase inhibitory potentials of a novel plant Olax nana. BMC Complement Altern Med.

[29] Tong X, Li X, Ayaz M, Ullah F, Sadiq A, Ovais M, et al. Neuroprotective studies on Polygonum hydropiper L. essential oils using transgenic animal models. Front Pharmacol. 2020;11. 10.3389/fphar.2020.580069 PMC7873646 33584260

[30] Saleem U, Akhtar R, Anwar F, Shah MA, Chaudary Z, Ayaz M, et al. Neuroprotective potential of Malva neglecta is mediated via down-regulation of cholinesterase and modulation of oxidative stress markers. Metab Brain Dis. 2021:1–12. 10.1007/s11011-021-00683-x 33570733

[31] Ovais M, Zia N, Ahmad I, Khalil AT, Raza A, Ayaz M, Sadiq A, Ullah F, Shinwari ZK (2018). Phyto-therapeutic and nanomedicinal approaches to cure Alzheimer’s disease: present status and future opportunities. Front Aging Neurosci.

[32] Ayaz M, Sadiq A, Junaid M, Ullah F, Ovais M, Ullah I, Ahmed J, Shahid M (2019). Flavonoids as prospective neuroprotectants and their therapeutic propensity in aging associated neurological disorders. Front Aging Neurosci.

[33] Mir NT, Saleem U, Anwar F, Ahmad B, Ullah I, Hira S, Ismail T, Ali T, Ayaz M (2019). Lawsonia Inermis markedly improves cognitive functions in animal models and modulate oxidative stress markers in the brain. Medicina.

[34] Sadiq A, Zeb A, Ullah F, Ahmad S, Ayaz M, Rashid U, Muhammad N (2018). Chemical characterization, analgesic, antioxidant, and anticholinesterase potentials of essential oils from Isodon rugosus Wall. ex. Benth. Front Pharmacol.

[35] Sadiq A, Rashid U, Ahmad S, Zahoor M, AlAjmi MF, Ullah R, Noman OM, Ullah F, Ayaz M, Khan I (2020). Treating hyperglycemia from eryngium caeruleum M. Bieb: In-vitro α-glucosidase, antioxidant, in-vivo antidiabetic and molecular docking-based approaches. Front Chem.

[36] Ayaz M, Junaid M, Ullah F, Sadiq A, Shahid M, Ahmad W, Ullah I, Ahmad A, Syed N-I-H (2017). GC-MS analysis and gastroprotective evaluations of crude extracts, isolated saponins, and essential oil from Polygonum hydropiper L. Front Chem.

[37] Shah S, Shah SMM, Ahmad Z, Yaseen M, Shah R, Sadiq A, Khan S, Khan B (2015). Phytochemicals, in vitro antioxidant, total phenolic contents and phytotoxic activity of Cornus macrophylla Wall bark collected from the North-West of Pakistan. Pak J Pharm Sci.

[38] Zeb A, Sadiq A, Ullah F, Ahmad S, Ayaz M (2014). Investigations of anticholinestrase and antioxidant potentials of methanolic extract, subsequent fractions, crude saponins and flavonoids isolated from Isodon rugosus. Biol Res.

[39] Amin MJ, Miana GA, Rashid U, Rahman KM, Khan H-U, Sadiq A (2020). SAR based in-vitro anticholinesterase and molecular docking studies of nitrogenous progesterone derivatives. Steroids.

[40] Sadiq A, Mahmood F, Ullah F, Ayaz M, Ahmad S, Haq FU, Khan G, Jan MS (2015). Synthesis, anticholinesterase and antioxidant potentials of ketoesters derivatives of succinimides: a possible role in the management of Alzheimer’s. Chem Cent J.

[41] Ahmad A, Ullah F, Sadiq A, Ayaz M, Jan MS, Shahid M, Wadood A, Mahmood F, Rashid U, Ullah R (2020). Comparative cholinesterase, α-glucosidase inhibitory, antioxidant, molecular docking, and kinetic studies on potent succinimide derivatives. Drug Des Devel Ther.

[42] Ghufran M, Rehman AU, Shah M, Ayaz M, Ng HL, Wadood A (2020). In-silico design of peptide inhibitors of K-Ras target in cancer disease. J Biomol Struct Dyn.

[43] Iftikhar F, Yaqoob F, Tabassum N, Jan MS, Sadiq A, Tahir S, Batool T, Niaz B, Ansari FL, Choudhary MI (2018). Design, synthesis, in-vitro thymidine phosphorylase inhibition, in-vivo antiangiogenic and in-silico studies of C-6 substituted dihydropyrimidines. Bioorg Chem.

[44] Tanoli ST, Ramzan M, Hassan A, Sadiq A, Jan MS, Khan FA, Ullah F, Ahmad H, Bibi M, Mahmood T (2019). Design, synthesis and bioevaluation of tricyclic fused ring system as dual binding site acetylcholinesterase inhibitors. Bioorg Chem.

[45] Bibi M, Qureshi NA, Sadiq A, Farooq U, Hassan A, Shaheen N, Asghar I, Umer D, Ullah A, Khan FA (2021). Exploring the ability of dihydropyrimidine-5-carboxamide and 5-benzyl-2, 4-diaminopyrimidine-based analogues for the selective inhibition of L. major Dihydrofolate reductase. Eur J Med Chem.

[46] Zahoor M, Shafiq S, Ullah H, Sadiq A, Ullah F (2018). Isolation of quercetin and mandelic acid from Aesculus indica fruit and their biological activities. BMC Biochem.

[47] Iqbala N, Ullaha F, Sadiqa A, Jana MS, Shahidb M, Ayaza M (2020). Cholinesterase’s enzymes inhibition and Michaelis-Menten kinetics studies on ethnomedicinally important plant chenopodium botrys. J Food Health Bioenviron Sci.

[48] Sultana N, Sarfraz M, Tanoli ST, Akram MS, Sadiq A, Rashid U, Tariq MI (2017). Synthesis, crystal structure determination, biological screening and docking studies of N1-substituted derivatives of 2, 3-dihydroquinazolin-4 (1H)-one as inhibitors of cholinesterases. Bioorg Chem.

[49] Ullah F, Ayaz M, Sadiq A, Hussain A, Ahmad S, Imran M, Zeb A (2016). Phenolic, flavonoid contents, anticholinesterase and antioxidant evaluation of Iris germanica var; florentina. Nat Prod Res.

[50] Chittaranjan Patra IA, Ayaz M, Khalil AT, Mukherjee S, Ovais M (2021). Biogenic nanoparticles for cancer theranostics.

[51] Khalil AT, Ovais M, Iqbal J, Ali A, Ayaz M, Abbas M, Ahmad I, Devkota HP (2021). Microbes-mediated synthesis strategies of metal nanoparticles and their potential role in cancer therapeutics. Seminars in cancer biology.

[52] Hussain F, Khan Z, Jan MS, Ahmad S, Ahmad A, Rashid U, Ullah F, Ayaz M, Sadiq A (2019). Synthesis, in-vitro α-glucosidase inhibition, antioxidant, in-vivo antidiabetic and molecular docking studies of pyrrolidine-2, 5-dione and thiazolidine-2, 4-dione derivatives. Bioorg Chem.

[53] Ahmad S, Ullah F, Ayaz M, Zeb A, Ullah F, Sadiq A (2016). Antitumor and anti-angiogenic potentials of isolated crude saponins and various fractions of Rumexhastatus D. Don. Biol Res.

[54] Bibi A, Shah T, Sadiq A, Khalid N, Ullah F, Iqbal A (2019). L-isoleucine-catalyzed michael synthesis of N-alkylsuccinimide derivatives and their antioxidant activity assessment. Russ J Org Chem.

[55] Shah SMM, Sadiq A, Shah SMH, Ullah F (2014). Antioxidant, total phenolic contents and antinociceptive potential of Teucrium stocksianum methanolic extract in different animal models. BMC Complement Altern Med.

[56] Jabeen M, Ahmad S, Shahid K, Sadiq A, Rashid U (2018). Ursolic acid hydrazide based organometallic complexes: synthesis, characterization, antibacterial, antioxidant, and docking studies. Front Chem.

[57] Menichini F, Losi L, Bonesi M, Pugliese A, Loizzo MR, Tundis R (2014). Chemical profiling and in vitro biological effects of Cardiospermum halicacabum L.(Sapindaceae) aerial parts and seeds for applications in neurodegenerative disorders. J Enzyme Inhib Med Chem.

[58] Wu T-Z (1999). A piezoelectric biosensor as an olfactory receptor for odour detection: electronic nose. Biosens Bioelectron.

[59] Laroze LE, Díaz-Reinoso B, Moure A, Zúñiga ME, Domínguez H (2010). Extraction of antioxidants from several berries pressing wastes using conventional and supercritical solvents. Eur Food Res Technol.

[60] Salem MZM, Abdel-Megeed A, Ali HM (2014). Stem wood and bark extracts of Delonix regia (Boj. Ex. Hook): Chemical analysis and antibacterial, antifungal, and antioxidant properties. BioResources.

[61] Smeriglio A, Alloisio S, Raimondo FM, Denaro M, Xiao J, Cornara L, Trombetta D (2018). Essential oil of Citrus lumia Risso: Phytochemical profile, antioxidant properties and activity on the central nervous system. Food Chem Toxicol.

[62] Hailu YM, Atlabachew M, Chandravanshi BS, Redi-Abshiro M (2017). Composition of essential oil and antioxidant activity of Khat (Catha edulis Forsk), Ethiopia. Chem Int.

[63] Tundis R, Bonesi M, Pugliese A, Nadjafi F, Menichini F, Loizzo MR (2015). Tyrosinase, acetyl-and butyryl-cholinesterase inhibitory activity of Stachys lavandulifolia Vahl (Lamiaceae) and its major constituents. Rec Nat Prod.

[64] Ruberto G, Baratta MT (2000). Antioxidant activity of selected essential oil components in two lipid model systems. Food Chem.

[65] Ya-Juan W, Xiao-Yan F, Ming B, Ming-San M. Study on the modern application of Schisandra Chinensis. DEStech Transactions on Biology and Health 2017(mshh).

[66] Demirel Z, Yilmaz-Koz FF, Karabay-Yavasoglu NU, Ozdemir G, Sukatar A (2011). Antimicrobial and antioxidant activities of solvent extracts and the essential oil composition of Laurencia obtusa and Laurencia obtusa var. pyramidata. Rom Biotechnol Lett.

[67] Park K-W, Kim D-Y, Lee S-Y, Kim J-H, Yang D-S (2011). A multivariate statistical approach to comparison of essential oil composition from three mentha species. Horticult Sci Technol.

[68] Chandra D, Chaubey P, Parki A, Prakash O, Kumar R, Pant A (2017). Study on chemical diversity among plant parts of Zingiber chrysanthum and their antioxidant assay. J Biol Active Prod Nat.

[69] Figueiredo AC (2017). Biological properties of essential oils and volatiles: sources of variability. Nat Volatiles Essent Oils.

[70] Chodaton-Zinsou MD, Assogba FM, Yayi-Ladékan E, Gbaguidi F, Moudachirou M, Gbénou JD (2020). Phytochemical composition, biological activities of croton lobatus L. leaves, hydrolysis effect on activities and chemical composition. Am J Appl Chem.

[71] Hadi MY, Mohammed GJ, Hameed IH (2016). Analysis of bioactive chemical compounds of Nigella sativa using gas chromatography-mass spectrometry. J Pharmacogn Phytother.

[72] Jisha M, Hukuman NZ, Leena P, Nair V (2021). Antioxidant, antimicrobial, anticorrosion and molecular docking studies on Alpinia calcarata Rosc., rhizome and leaf extracts.

[73] Ahmad S, Ullah F, Sadiq A, Ayaz M, Imran M, Ali I, Zeb A, Ullah F, Shah MR (2016). Chemical composition, antioxidant and anticholinesterase potentials of essential oil of Rumex hastatus D. Don collected from the North West of Pakistan. BMC Complement Altern Med.

[74] Saleh IA, Usman K, Abu-Dieyeh MH (2020). Halophytes as important sources of antioxidants and anti-cholinesterase compounds. Handbook of halophytes: from molecules to ecosystems towards biosaline agriculture.

[75] da Silva JKR, Pinto L, Burbano R, Montenegro RC, Guimarães EF, Andrade EHA, Maia JGS (2014). Essential oils of Amazon piper species and their cytotoxic, antifungal, antioxidant and anti-cholinesterase activities. Ind Crop Prod.

