# Supplementary material for: Neuroprotective potentials of selected natural edible oils using enzyme inhibitory, kinetic and simulation approaches
Source: BMC Complement Med Ther. 2021 Oct 2;21:248. doi: 10.1186/s12906-021-03420-0 (PMC8487577; doi:10.1186/s12906-021-03420-0)
Supplement: Supplementary file 1 — Additional file 1: Table S1. Details of identified compounds in GC-MS analysis of cinnamon oil. Table S2. Chemical composition of ginger essential oil. Table S3. Chemical composition of cumin essential oil. Table S4. Antioxidant of the selected essential oils using ascorbic acid as standard. [file 12906_2021_3420_MOESM1_ESM.docx]

**Neuroprotective potentials of selected natural edible oils using enzyme inhibitory, kinetic and simulation approaches**

Mater H. Mahnashi^1^ ([matermaha@gmail.com](mailto:matermaha@gmail.com))

Bandar A. Alyami^1^ ([alyamibandar1@gmail.com](mailto:alyamibandar1@gmail.com))

Yahya S. Alqahtani^1^ ([yahyasalqahtani0@gmail.com](mailto:yahyasalqahtani0@gmail.com))

Ali O. Alqarni^1^ ([aoqarni@gmail.com](mailto:aoqarni@gmail.com))

Muhammad Saeed Jan^2^ ([saeedjanpharmacist@gmail.com](mailto:saeedjanpharmacist@gmail.com))

Muhammad Ayaz^3^ ([ayazuop@gmail.com](mailto:ayazuop@gmail.com))

Farhat Ullah^3^ ([farhataziz80@hotmail.com](mailto:farhataziz80@hotmail.com))

Muhammad Shahid^4^ ([shahidsalim_2002@hotmail.com](mailto:shahidsalim_2002@hotmail.com))

Umer Rashid^5^ ([umerrashid@cuiatd.edu.pk](mailto:umerrashid@cuiatd.edu.pk))

Abdul Sadiq*^3^ ([sadiquom@yahoo.com](mailto:sadiquom@yahoo.com))

^1^Department of Pharmaceutical Chemistry, College of Pharmacy, Najran University, Najran, Saudi Arabia. ^2^Department of Pharmacy, University of Swabi, KP, Pakistan. ^3^Department of Pharmacy, Faculty of Biological Sciences, University of Malakand, Chakdara, 18000 Dir (L), KP, Pakistan. ^4^Department of Pharmacy, Sarhad University of Science and Information Technology, Peshawar 25000, Khyber Pakhtunkhwa, Pakistan. ^5^Department of Chemistry, COMSATS University Islamabad, Abbottabad Campus, 22060 Abbottabad, Pakistan.

**Corresponding author:** *Dr. Abdul Sadiq, Associate Professor, Department of Pharmacy, University of Malakand, Chakdara, 18000 Dir (L), KP, Pakistan, Contact: +92(0)301 2297 102, Email: [sadiquom@yahoo.com](mailto:sadiquom@yahoo.com).

**Supporting Information**

**Table S1:** Details of identified compounds in GC-MS analysis of cinnamon oil

| S.NO | Compound Label | RT | Common Name | Formula | Hits (DB) |
| --- | --- | --- | --- | --- | --- |
| 1 | Quadricyclanone | 3.6 | Quadricyclanone | C_7_H_6_O | 8 |
| 2 | 1,2,2,5-Tetramethyl-3-oxo-4-hexenyl Acetate | 3.908 | (Z)-hexenyl Acetate | C_12_H_20_O_2_ | 10 |
| 3 | Heptenal | 4.681 | heptenal | C_7_H_12_O | 10 |
| 4 | Caproic acid | 5.009 | Hexanoic acid | C_6_H_12_O_2_ | 10 |
| 5 | 2,4-Decadien-1-al | 9.826 | 2,4-Decadienal | C_10_H_16_O | 10 |
| 6 | 10-Methylnonadecane | 10.186 | 10-Methylnonadecane | C_20_H_42_ | 10 |
| 7 | Trans-Caryophyllene | 11.667 | Caryophyllene | C_15_H_24_ | 10 |
| 8 | BETA-Chamigrene | 12.07 | Chamigrene | C_15_H_24_ | 10 |
| 9 | -(-)Alloaromadendren | 12.426 | Alloaromadendren | C_15_H_24_ | 10 |
| 10 | Beta-Himachalene | 12.696 | Beta-Himachalene | C_15_H_24_ | 10 |
| 11 | 4,6-Dimethyldodecane | 12.988 | dodecane | C_14_H_30_ | 10 |
| 12 | (E)-1,2,4,4-tetramethyl-3-(3'-methyl-1',3'-butadienyl)-2-cyclohexen-1-ol | 13.135 | N/A | C_15_H_24_O | 5 |
| 13 | 1,5-Cyclooctadiene, 3-t-butyl- | 14.07 | N/A | C_12_H_20_ | 10 |
| 14 | Italicene | 14.537 | italicene | C_15_H_24_ | 10 |
| 15 | 2-Isopropyl-5-methyl-1-heptanol | 15.927 | N/A | C_11_H_24_O | 10 |
| 16 | Alpha Atlantone | 16.222 | Alpha Atlantone | C_15_H_22_O | 10 |
| 17 | Hexadecanoic acid | 20.249 | Palmitic acid | C_16_H_32_O_2_ | 10 |
| 18 | Cis-9, cis-12-Octadecadienoic acid | 26.854 | 9,12 -Octadecadienoic acid | C_18_H_32_O_2_ | 10 |
| 19 | 3,5-dimethyl-4-aza-4-heptene | 26.987 | Butanamine | C_8_H_17_N | 3 |
| 20 | 3-Decyn-1-ol | 27.375 | N/A | C_10_H_18_O | 6 |
| 21 | n-tetracosane | 28.066 | Tetracosaine | C_24_H_50_ | 10 |
| 22 | 4-(2-Furyl)-3-butanone | 32.826 | N/A | C_8_H_10_O_2_ | 1 |
| 23 | 2-hydroxy-1-(hydroxymethyl) ethyl ester | 33.869 | 9,12-octadecadienoic acid | C_21_H_38_O_4_ | 2 |
| 24 | 1H-indole, 3 methyl- | 34.961 | Skatole | C_9_H_9_N | 10 |
| 25 | Cyclohexyl dimethyl silyloxy butane | 38.02 | butyldimethylsiloxycyclohexane | C_12_H_26_OSi | 10 |
| 26 | 1-[5-(1,3-benzodioxol-5-yl)-1-oxo-2,4-pentadienyl]-, (E,E)- | 39.688 | Piperidine | C_17_H_19_NO_3_ | 10 |
| 27 | Adipic acid | 40.357 | Propyl ester | C_14_H_25_NO_3_ | 4 |
| 28 | 4,5-Dimethyl-3-(p tolylsulfinyl)hexan-2-one | 40.422 | N/A | C_15_H_22_O_2_S | 1 |
| 29 | 7-Amino-5-bromo-8-cyano-2-methyl-1,6-naphthyridine | 43.047 | N/A | C_10_H_7_BrN_4_ | 10 |
| 30 | (2R*,3aS*,5R*,6aR*)-2,5-Di(tert-butyl)-2,5-dimethylperhydrofuro[2,3-b]furan | 49.047 | N/A | C_16_H_30_O_2_ | 4 |
| 31 | 2,2,5,5-Tetramethyl-3-(p-nitrophenyl)pyrroliden-1-oxyl | 52.592 | N/A | C_14_H_19_N_2_O_3_ | 1 |
| 32 | (2,3-Dimethoxyphenyl)hydrazine | 56.988 | (2,3-Dimethoxyphenyl)hydrazine) | C_8_H_12_N_2_O_2_ | 1 |
| 33 | Monoacetylhydrazone of 9,10-phenanthraquinone | 64.894 | N/A | C_16_H_12_N_2_O_2_ | 4 |
| 34 | N-benzoylbenzimidsaure-isopropylester | 74.745 | N/A | C_17_H_17_NO_2_ | 1 |

**Table S2: chemical composition of ginger essential oil.**

| S. No | Compound Label | RT | Common Name | formula | Hits (DB) |
| --- | --- | --- | --- | --- | --- |
| 1 | 1,7-Octadiene, 2,3,3-trimethyl- | 3.695 | N/A | C_11_H_20_ | 10 |
| 2 | 1-Hexadecanol, 3,7,11,15-tetramethyl- | 26.377 | Dihydrophytol | C_20_H_42_O | 10 |
| 3 | 4,8,12-Trimethyltridecan-4-olide | 46.269 | N/A | C_16_H_30_O_2_ | 10 |
| 4 | N-[(E)-2-Methyl-3-{3,5-dimethoxyphenyl}-2-propenoyl]bornane-10,2-sultam | 54.573 | 2-sultam | C_22_H_29_NO_5_S | 1 |
| 5 | 3-Diphenylphosphino-4,6-diphenyl-.lamda.(3)-phosphinine | 56.223 | N/A | C_29_H_22_P_2_ | 3 |
| 6 | 1-Oxo-2(1H)-isoquinolineacetic Acid | 57.519 | N/A | C_11_H_9_NO_3_ | 10 |
| 7 | 2,3-Bis(morpholinomethylene)bicyclo[2.2.1]hepta-2,5-diene | 57.742 | N/A | C_17_H_26_N_2_O_2_ | 3 |
| 8 | 2-Cyano-1-(pyrrolidin-2'-yl)-pyrrolidine | 59.017 | N/A | C_9_H_15_N_3_ | 10 |
| 9 | Decyl pivalate | 59.631 | Decyl pivalate | C_15_H_30_O_2_ | 6 |
| 10 | O-Diacryloyl-cis,cis-spiro[4.4]nonane-1,6-diol | 59.683 | N/A | C_15_H_20_O_4_ | 1 |
| 11 | (3S,3aS,4S,5S,7S,7aS)-7-Iodo-4,5-di(4-methoxybenzyl)oxy-3-methyl-3a,4,6,6,7,... | 59.782 | N/A | C_25_H_29_IO_6_ | 10 |
| 12 | (22R,23R,24R)-3.alpha-Bromo-2-.beta.,22,23-trihydroxy-24-methyl-5.alpha.-cho... | 60.52 | N/A | C_28_H_47_BrO_4_ | 2 |
| 13 | 5-(4-Methoxyphenyl)-7-benzyl-1,11,11-trimethyl-5-aza-2-oxatetracyclo[6.5.0.0... | 61.115 | N/A | C_28_H_33_NO_3_ | 9 |
| 14 | Isoquinoline, 1,2,3,4-tetrahydro-1,2-dimethyl- | 63.569 | N/A | C_11_H_15_N | 10 |

**Table S3: chemical composition of cumin essential oil.**

| S.NO | Compound Label | RT | Common Name | formula | Hits (DB) |
| --- | --- | --- | --- | --- | --- |
| 1 | 4-Cycloocten-1-yl methyl ether | 3.744 | 5-Methoxy-cyclooctene | C_9_H_16_O | 7 |
| 2 | 5-Methylhexan-1-al | 3.901 | 5-Methylhexanal | C_7_H_14_O | 10 |
| 3 | Diethyl 1-(ethoxycarbonyl)-1-hydroxyethanephophonate | 4.403 | N/A | C_9_H_19_O_6_P | 1 |
| 4 | heptenal | 4.723 | Heptenal | C_7_H_12_O | 10 |
| 5 | n-Oct-1-en-3-ol | 5.07 | Hydroxy-1-octene | C_8_H_16_O | 10 |
| 6 | n-Hexanoic acid | 5.187 | Caproic acid | C_6_H_12_O_2_ | 10 |
| 7 | 2-(3,3-Dimethylbutyl)oxirane | 5.401 | Oxirane | C_8_H_16_O | 10 |
| 8 | 1-Nonen-4-ol | 5.574 | N/A | C_9_H_18_O | 10 |
| 9 | OCTENAL | 6.31 | OCTENAL | C_8_H_14_O | 10 |
| 10 | n-Nonylaldehyde | 7.024 | Nonanal | C_9_H_18_O | 10 |
| 11 | cis-1,1,3,4-Tetramethylcyclopentane | 7.898 | Cyclopentane | C_9_H_18_ | 10 |
| 12 | n-Octoic acid | 8.184 | Octanoic Acid | C_8_H_16_O_2_ | 10 |
| 13 | 7-Methyl-5-undecene | 8.319 | 5-methylundecane | C_12_H_24_ | 10 |
| 14 | 8-Methyl-1-undecene | 9.173 | N/A | C_12_H_24_ | 10 |
| 15 | 2-Propenal, 3-phenyl- | 9.653 | Cinnamal | C_9_H_8_O | 10 |
| 16 | ,4-Decadien-1-al | 9.886 | 2,4-Decadienal | C_10_H_16_O | 10 |
| 17 | (S)-2-Acetyl-2-cyclopenten-1-ol | 10.604 | N/A | C_7_H_10_O_2_ | 10 |
| 18 | 1,2-Dimethyl-1-cyclodecene | 10.657 | Cyclododecene | C_12_H_22_ | 10 |
| 19 | 3,3,4,4-Tetramethyl-2-pentanone | 10.75 | N/A | C_9_H_18_O | 10 |
| 20 | Undecenoic aldehyde | 10.831 | Undecenal | C_11_H_20_O | 10 |
| 21 | 2-Methyl-1-cyclohexanone | 11.055 | 2-methyl cyclohexanone | C_7_H_12_O | 10 |
| 22 | 4,6-Dimethylundecane | 12.537 | N/A | C_13_H_28_ | 10 |
| 23 | trans-4-Methoxycinnamaldehyde | 13.114 | N/A | C_10_H_10_O_2_ | 10 |
| 24 | 8-Hexadecyne | 14.623 | 8-Hexadecyne | C_16_H_30_ | 10 |
| 25 | 8-Heptadecene | 14.706 | 8-Heptadecene | C_17_H_34_ | 10 |
| 26 | Hexadecanoic acid | 20.666 | Palmitic acid | C_16_H_32_O_2_ | 10 |
| 27 | 6,7-Dimethoxybenzo[c][1,4]-oxathiaocane 5,8-dione | 20.859 | N/A | C_11_H_12_O_5_S | 1 |
| 28 | (Z)-6-(Tetrahydropyran-2-yloxy)-1-trimethylsilylhex-2-ene | 25.685 | N/A | C_14_H_28_O_2_Si | 1 |
| 29 | (1S,7aS)-1-Methoxymyodesertan | 26.53 | N/A | C_11_H_20_O_2_ | 10 |
| 30 | Methylene cyclohexane | 27.451 | Methylene cyclohexane | C_7_H_12_ | 10 |
| 31 | cis-9-Tricosene | 27.582 | Muscalure | C_23_H_46_ | 10 |
| 32 | 3-(2-Oxocyclohexyl)propionaldehyde | 29.45 | 3-(2-oxocyclohexyl)propanoic acid | C_9_H_14_O_2_ | 2 |
| 33 | Cyclononene, 3-(2-propenyl) | 29.531 | N/A | C_12_H_20_ | 1 |
| 34 | 1-oxa-2-cyano-6-methyl-cyclohex-2-ene | 31.438 | N/A | C_7_H_9_NO | 10 |
| 35 | Hexane-2,5-dione | 32.597 | 1,2-Diacetylethane | C_6_H_10_O_2_ | 1 |
| 36 | Oxacyclononadec-10-en-2-one | 34.652 | N/A | C_18_H_32_O_2_ | 10 |
| 37 | 1,2-Benzenedicarboxylic acid, bis(2-ethylhexyl) ester | 36.43 | DNOP | C_24_H_38_O_4_ | 10 |
| 38 | nor-phytane | 37.196 | Nonadecane | C_19_H_40_ | 10 |
| 39 | 1,3-Dithiane, 2-phenyl- | 38.209 | 2-Phenyl-m-dithiane | C_10_H_12_S_2_ | 1 |
| 40 | 1,3-Dideutero-isoquinoline | 38.27 | N/A | C_9_H_5_D_2_N | 2 |
| 41 | Sulfurous acid, pentyl tridecyl ester | 38.967 | N/A | C_18_H_38_O_3_S | 10 |
| 42 | Bicyclo[2.2.1]heptan-2-one, 1,7,7-trimethyl- | 39.219 | CAMPHORE | C_10_H_16_O | 5 |
| 43 | 9,12-Octadecadienoic acid (Z,Z)-, 2-hydroxy-1-(hydroxymethyl)ethyl ester | 39.466 | 2-Monolinolein | C_21_H_38_O_4_ | 2 |
| 44 | (Z,Z)-7,15-Tetracosadiene-1,24-diol | 39.55 | N/A | C_24_H_46_O_2_ | 10 |
| 45 | Decane, 1,1'-thiobis- | 40.011 | Decyl sulfide | C_20_H_42_S | 2 |
| 46 | n - tri - tria - contanene | 40.498 | Tritriacontane | C_33_H_68_ | 10 |
| 47 | Hexadecane, 2,6,10,14-tetramethyl- | 41.949 | Phytan | C_20_H_42_ | 10 |
| 48 | Papaverine | 42.191 | N/A | C_20_H_21_NO_4_ | 10 |
| 49 | 3-Pentyltetrahydrothiophene 1,1-dioxide | 42.749 | Sulfolane | C_9_H_18_O_2_S | 2 |
| 50 | n-Docosane | 43.287 | Docosane | C_22_H_46_ | 10 |
| 51 | Pentadecanal- | 43.875 | Pentadecanal- | C_15_H_30_O | 10 |
| 52 | Isohexadecane | 44.684 | Hexadecane | C_16_H_34_ | 10 |
| 53 | 1-Docosanol | 45.254 | Behenic alcohol | C_22_H_46_O | 4 |
| 54 | 3-methyl-, 3,7-dimethyl-6-octenyl ester | 46.993 | Butanoic acid | C_15_H_28_O_2_ | 4 |
| 55 | 2-Benzylidene-benzofuran-3(2H)-one | 49.744 | Aurone | C_15_H_10_O_2_ | 10 |
| 56 | 4-(tert-Butyldimethylsilyloxy)-1-(p-toluenesulfonyl)-1-heptyne | 73.162 | N/A | C_17_H_26_O_3_SSi | 4 |

**Table S4: Antioxidant of the selected essential oils using ascorbic acid as standard.**

| Samples | Conc. (μg/mL) | ABTS | | DPPH | |
| --- | --- | --- | --- | --- | --- |
|  |  | Percent inhibition  (mean ± SEM) | IC_50_  (μg/ml) | Percent inhibition  (mean ± SEM | IC_50_  (μg/ml) |
| Cinnamon | 1000  500  250  125  62.5 | 74.40 ± 1.10***  66.91 ± 1.09***  53.78 ± 1.03***  51.57 ± 0.59***  47.85 ± 2.10*** | 93 | 69.06 ± 1.02***  67.92 ± 1.20***  61.99 ± 0.94***  53.42 ± 1.28***  47.37 ± 0.62*** | 85 |
| Ginger | 1000  500  250  125  62.5 | 73.65 ± 0.85***  64.44 ± 1.33***  57.02 ± 1.49***  54.56 ± 0.94***  47.83 ± 2.14*** | 77 | 71.75 ± 0.91***  62.66 ± 0.86***  52.44 ± 1.37***  50.68 ± 1.54***  46.51 ± 0.74*** | 121 |
| cumin | 1000  500  250  125  62.5 | 66.17 ± 1.79***  59.89 ± 2.04***  49.33 ± 2.33***  48.59 ± 3.21***  39.13 ± 1.43*** | 271 | 63.34 ± 0.72***  57.32 ± 1.29***  49.13 ± 0.83***  45.14 ± 2.08***  38.85 ± 1.02*** | 280 |
| Ascorbic acid | 1000  500  250  125  62.5 | 91.69 ± 0.42  87.63 ± 0.32  79.32 ± 1.21  70.19 ± 0.77  64.73 ± 1.19 | 08 | 90.61 ± 0.43  87.34 ± 0.38  78.67 ± 0.89  71.89 ± 0.65  64.96 ± 1.07 | 06 |

The values are presented as mean ± SEM (𝑛 = 3). The asterisk shows that the significance levels in comparison with that of the positive control: Data were analyzed via TWO-WAY ANOVA followed by Bonferroni post-test ^∗^ 𝑃 < 0.05; ^∗∗^ 𝑃 < 0.01, ^∗∗∗^ 𝑃 < 0.001, *ns;* P > 0.05.
